# New roles for old enzymes: killer caspases as the engine of cell behavior changes

**DOI:** 10.3389/fphys.2014.00149

**Published:** 2014-04-16

**Authors:** Patrick F. Connolly, Richard Jäger, Howard O. Fearnhead

**Affiliations:** ^1^Pharmacology and Therapeutics, National University of Ireland GalwayGalway, Ireland; ^2^Department of Natural Sciences, Bonn-Rhein-Sieg University of Applied SciencesRheinbach, Germany

**Keywords:** caspase, apoptosis, myogenesis, proliferation, differentiation, non-apoptotic roles

## Abstract

It has become increasingly clear that caspases, far from being merely cell death effectors, have a much wider range of functions within the cell. These functions are as diverse as signal transduction and cytoskeletal remodeling, and caspases are now known to have an essential role in cell proliferation, migration, and differentiation. There is also evidence that apoptotic cells themselves can direct the behavior of nearby cells through the caspase-dependent secretion of paracrine signaling factors. In some processes, including the differentiation of skeletal muscle myoblasts, both caspase activation in differentiating cells as well as signaling from apoptotic cells has been reported. Here, we review the non-apoptotic outcomes of caspase activity in a range of different model systems and attempt to integrate this knowledge.

## Introduction

Caspases are intracellular cysteine proteases that cut after specific aspartic acid residues. In mammals, 18 caspases have been characterized (Table 1), the majority of them playing roles in mediating apoptotic cell-demolition (Cohen, 1997). During apoptosis, these “killer caspases” cleave numerous cellular proteins, and they are the primary effectors responsible for taking apart the cell during apoptosis, playing a major proteolytic role in the disassembly of the nucleus and the cytoskeletal structure (Lüthi and Martin, 2007).

**Table 1:** **Roles of caspases**.

**Caspase**	**Conventional role**	**Other roles**
Caspase-1	Inflammatory response (Kuranaga and Miura, 2007)	Unknown
Caspase-2	Apoptosis (Initiator) (Inoue et al., 2009)	DNA damage response (Fava et al., 2012)
Caspase-3	Apoptosis (Executioner) (Kuranaga and Miura, 2007)	Differentiation of many cell types (see Table 2)
Caspase-5	Inflammatory response (Fuchs and Steller, 2011)	Possibly tumor suppression (Soung et al., 2008)
Caspase-6	Apoptosis (Initiator) (Inoue et al., 2009)	Unknown
Caspase-7	Apoptosis (Executioner) (Inoue et al., 2009)	Unknown
Caspase-8	Apoptosis (Executioner) (Fuchs and Steller, 2011)	Embryonic development (Suzanne and Steller, 2013), motility (Helfer et al., 2006), tumor metastasis (Barbero et al., 2009), T-cell proliferation (Kennedy et al., 1999), cell cycle regulation (Zhang et al., 2001; Hashimoto et al., 2011), bacterial infection response (Kayagaki et al., 2011)
Caspase-9	Apoptosis (Initiator) (Cohen, 1997)	Differentiation of many cell types (see Table 2)
Caspase-10	Apoptosis (Initiator) (Inoue et al., 2009)	Immune response to dsRNA (Takahashi et al., 2006), possible tumor suppressor (Park et al., 2002)
Caspase-11	Inflammatory response (Li et al., 2007)	Cell migration (Li et al., 2007)
Caspase-12	Inflammatory response (Leulier et al., 2000)	Unknown
Caspase-14	Keratinocyte differentiation (Zermati et al., 2001)	Unknown
Caspase-15	Apoptosis (Initiator) (Eckhart et al., 2005)	Unknown
Caspase-16	Unknown, phylogenetic association with caspase-14 (Eckhart et al., 2008)	Unknown
Caspase-17	Unknown, phylogenetic association with caspase-3 (Eckhart et al., 2008)	Unknown
Caspase-18	Unknown, phylogenetic association with caspase-8 (Eckhart et al., 2008)	Unknown

The majority of caspases are primarily involved in programmed cell death, and the minority are primarily involved in the generation of immune responses. Most have been discovered to play other roles as well.

Caspases are present in all cells as inactive zymogens, called procaspases. They are activated through cleavage to generate the subunits that form an active caspase (Pop and Salvesen, 2009). At the apex of the activation cascade are the so-called initiator caspases (Table 1). Upon exposure to an apoptotic stimulus they become recruited to specific adaptor proteins which then assemble into activation platforms, which are large multimeric protein complexes mediating activation of the initiator caspases (reviewed in Mace and Riedl, 2010). Initiators activate downstream effectors which rapidly disassemble the cell. The ability of these caspases to kill cells is controlled, in part, by the inhibitor of apoptosis proteins (IAPs) which bind to active caspases and either inhibit proteolytic activity or induce ubiquitin-mediated caspase degradation (Mace et al., 2010).

Different activation platforms classify the main apoptotic pathways. The extrinsic pathway is initiated at the cell membrane by ligands of receptors of the tumor necrosis factor (TNF) receptor family. Ligand-binding leads to assembly of the death-inducing signaling complex (DISC) containing these receptors. Caspase-8 and -10 are recruited to the DISC and then activated, in a process that requires the adaptor protein FADD. The intrinsic pathway is initiated by mitochondria, whose outer membranes become permeable to cytochrome *c* upon certain cellular stresses. Released cytochrome *c* then binds to the adaptor protein APAF-1 which subsequently assembles into a large heptameric protein complex, the so-called apoptosome, which is the activation platform for caspase-9. The release of cytochrome *c* is controlled by proteins of the Bcl-2 family (Tait and Green, 2010). Concomitant with release of cytochrome *c*, other small proteins may be released, some of which block IAPs allowing for unrestrained caspase activation.

The majority of studies of caspases have focused on their roles as cell killers. There were some notable exceptions describing caspase-dependent cellular differentiation processes that involve denucleation or other degenerative events (Fernando and Megeney, 2007), but these appear to represent a limited or frustrated apoptosis, rather than a fundamentally different process. More recently, the study of apoptotic caspases has broadened to include caspase-dependent paracrine signaling from apoptotic cells to explain how apoptotic cells alter the behavior of surrounding cells (Li et al., 2010a).

However, it has also emerged that apoptosis-associated caspases play non-apoptotic roles and that they are not simply destructive. Examples of these are cell differentiation (Fernando and Kelly, 2002), embryonic development (Miura, 2012; Suzanne and Steller, 2013), motility (Barbero et al., 2009), and compensatory proliferation (Fan and Bergmann, 2008). Within these processes, the “killer” caspases clearly do not cause cell demolition. This gives caspases an entirely new role in determining the fate or behavior of cells. Thus, caspases drive in a far wider range of cellular behaviors than previously known.

**Figure 1:**
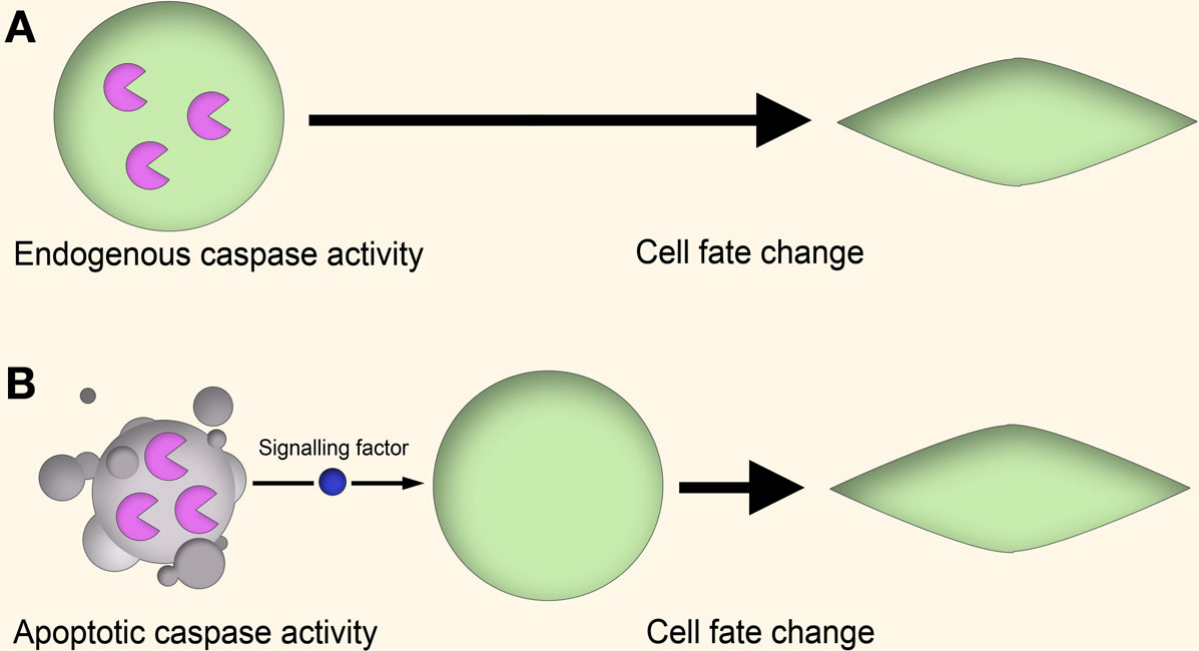
**Cell-autonomous vs. non-cell-autonomous models of caspase signaling. (A)** Cell-autonomous model. In the proliferating cell, a non-apoptotic caspase signaling pathway leads to a change in cell behavior through modulation of gene expression, cytoskeletal restructuring, or other means. This signaling is endogenous to the proliferating cell itself, and no apoptotic cell signaling is involved. **(B)** Non-cell-autonomous model. In this model, caspase activity within apoptotic cells lead to the generation of signaling factors which stimulate the cell behavior change of nearby cells in a paracrine fashion.

There are several theories to explain how apoptotic caspases can lead to non-apoptotic outcomes (Figure 1). In one mechanism, the “cell-autonomous” or “direct” model, caspase activity leads to altered cell behavior through the modulation of regulatory networks, such as through cleavage of cell cycle repressors to alter cell proliferation (Schwerk and Schulze-Osthoff, 2003; Woo et al., 2003), activation of gene transcription to induce skeletal muscle differentiation (Larsen and Megeney, 2010) or cleavage of cytoskeletal proteins to influence cell motility (Helfer et al., 2006). In these cases the caspase activity is “autonomous” in that the entire process of caspase activation, cleavage of substrates, and downstream effects on cell behavior all occur within the same cell.

In this cell autonomous model, it is as yet unclear how apoptotic caspase activity is harnessed for non-apoptotic purposes without killing the cell, although work in *Drosophila melanogaster* has begun to unravel this problem. For example, a recent report of caspase activation in *Drosophila* proposes a model in which both the magnitude and rate of caspase activation is controlled, which can give rise to high (apoptotic) levels of caspase activity as well as low (non-apoptotic) levels of activity (Florentin and Arama, 2012). It is also possible that, unlike the traditional model where executioner caspases are only activated upon receipt of a cell stress signal, there is a constant basal level of activated caspases within the cell, but these are normally held in check by inhibitory mechanisms. Such basal levels of caspase activity have been found in the context of cell behavior changes in glioblastoma cells, where low levels of constitutively-active caspase-8 and -3 are found to be necessary for cell migration and invasion (Gdynia et al., 2007). Along with this, relatively high levels of caspases activity may be tolerated if they can be sequestered within their target organelle or sub-cellular region, as is observed in the dendritic pruning of neurons (Williams et al., 2006), in spermatid individualization in Drosophila (Arama et al., 2007; Kaplan et al., 2010), and in the nuclear degradation of keratinocytes (Weil et al., 1999).

In the “non-autonomous” or “indirect” model to explain the role of caspases in non-apoptotic processes, the caspase activity is localized within apoptotic cells, catalyzing the generation of secretory paracrine signaling factors or enabling cell surface-mediated signaling (Hochreiter-Hufford et al., 2013). This model is indirect in that the caspase activity is associated with one cell, while the downstream effect is induced in another cell by an inter-cellular signaling event. In this model the caspase-mediated non-apoptotic effects do not necessarily require the survival of the “caspase-active” cell, as apoptotic cells are still quite capable of signaling to their environment (Jäger and Fearnhead, 2012).

Here, we review the major non-apoptotic roles of caspases discovered to date, and discuss these findings in light of the direct and indirect theories of caspase signaling, with a particular focus on skeletal muscle. This is a rapidly advancing field of study, and a summation of the current state of the field is necessary.

## Tissue repair and regeneration

**Figure 2:**
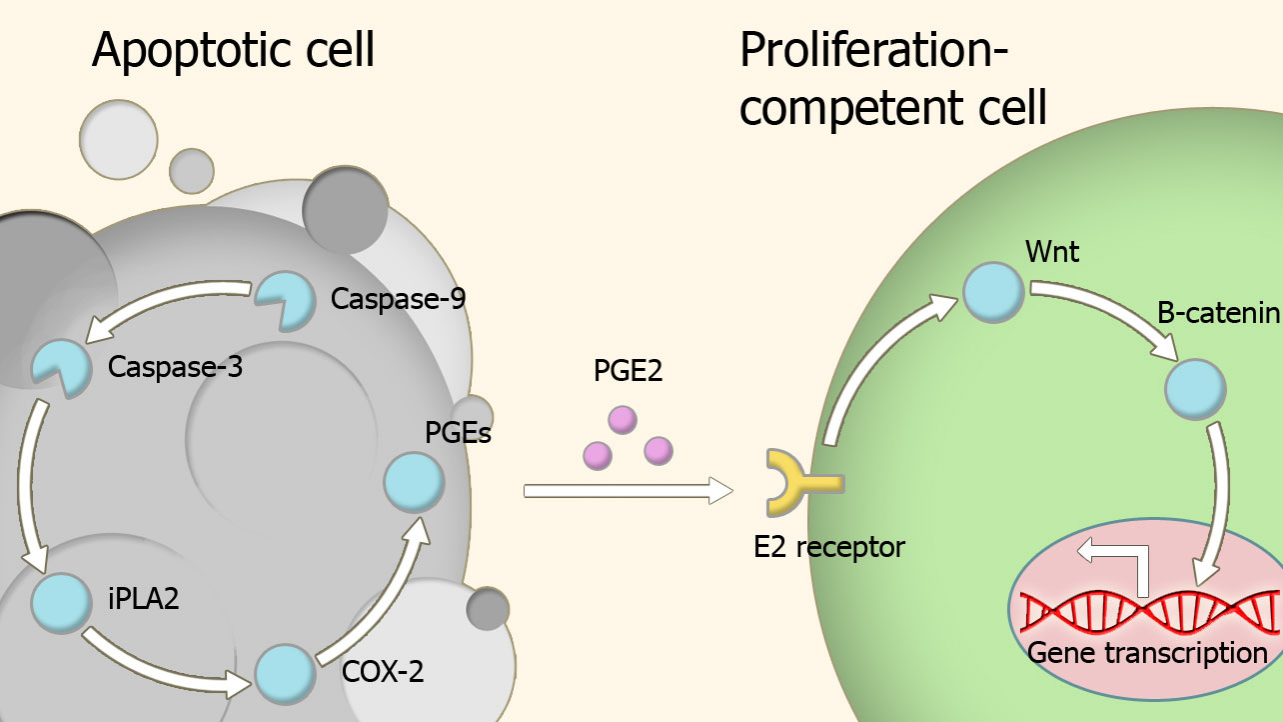
**Model of compensatory proliferation.** Caspase activity within apoptotic cells leads to the activation of the prostaglandin E2-synthesis pathway. Secreted prostaglandin E2 binds to E2 receptors on proliferation-competent cells, leading to changes in gene expression which trigger proliferation. Abbreviations: iPLA2, Phospholipase A2; COX-2, Cyclooxygenase-2; PGEs, Prostaglandin E synthase; PGE2, Prostaglandin E2.

Caspases are key players in the homeostatic balance between apoptosis and regeneration used to maintain tissue structure and function. In response to injury, dead cells engage in a signaling behavior which drives the proliferation of cells at the periphery of the site of injury until damaged portion of tissue is replaced with a new section of the same size and shape (Figure 2) (Bergmann and Steller, 2010). The role of caspases in repair and regeneration has been demonstrated in several different experimental models.

In the simple metazoan Hydra, surgical-induced injury produces an apoptotic response which stimulates a compensatory proliferative mechanism in surrounding progenitor cells. Treatment with pan-caspase inhibitors abolishes this regenerative response (Cikala et al., 1999; Chera et al., 2009). Regeneration in the amphibian *Xenopus* requires caspase-mediated events (Tseng et al., 2007), as does tissue regeneration in planaria (Fuchs and Steller, 2011), and the regeneration of newt forelimbs (Vlaskalin et al., 2004).

Regeneration of mammalian tissue is never so dramatic but some tissues, like the liver, can undergo remarkable regeneration after injury (Taub, 2004). Liver regeneration and the healing of skin wounds is impaired in caspase-3 and -7 deficient mice, showing that the role of caspases in regenerative processes is conserved in mammals (Li et al., 2010a).

Paracrine molecules secreted by the apoptotic cells appear to be important in caspase-dependent regeneration. Prostaglandin E2 is one such a molecule, as its production pathway is directly controlled by caspase-3 (Boland et al., 2013), and has a wide number of roles in regeneration and proliferation (Castellone et al., 2005; Goessling et al., 2009; Morata et al., 2011; Beaulieu et al., 2012; Boland et al., 2013). This effect is exerted through transient activation of the Wnt-β-catenin pathway via binding to members of the EP receptor family (Goessling et al., 2009).

Lysophosphatidylcholine (LPC) is another molecule which mediates caspase-activity-induced regenerative responses. It is produced by apoptotic cells, and its presence in the interstitial medium acts as an attraction signal to phagocytes (Lauber et al., 2003). Moreover, LPC induces the differentiation of keratinocytes, which is a necessary step in the wound healing response in skin (Ryborg et al., 2004).

Sphingosine-1-phosphate is another molecule that is secreted by apoptotic cells and is a chemoattractant signal for immune cells (Brecht et al., 2011). It is produced by the enzyme ceramidase, and is a signal that drives growth arrest and differentiation, as well as cell migration and adhesion (Mao and Obeid, 2008), all of which are involved in wound-healing responses.

Fractalkine (CX3CL1) is a large peptide that engages in pro-survival functions in many cell types (White and Greaves, 2012) and is released from apoptotic cells in a caspase-dependant process (Truman et al., 2008). Fractalkine is normally associated with immune cell chemotaxis (Chazaud et al., 2003), but it is also known that soluble fractalkine promotes both migration of endothelial cells and differentiation of osteoblasts (Koizumi et al., 2009). This makes it another potential paracrine signaling factor released by apoptotic cells to modulate tissue regeneration.

From the examples described, it is seen that caspase activity in a dying cell can indirectly induce compensatory proliferation of neighboring cells as part of the regenerative response to injury. Thus, regenerative processes conform to the indirect, apoptotic cell-driven model of caspase function in non-apoptotic processes. However, caspases can also regulate cell proliferation in a cell-autonomous manner through the cleavage of cell cycle regulators.

## Lymphocyte proliferation

There are hundreds of confirmed caspase substrates (Lüthi and Martin, 2007; Johnson and Kornbluth, 2008), although the functional significance of cleavage is often uncertain. Among this large group there are several key cell cycle regulators (Hashimoto et al., 2011) and a series of studies have implicated both initiator and effector caspases in the control of the cell cycle of lymphocytes. Because of this, abnormal caspase activity can lead to either depressed or hyperactive cell proliferation.

Through the DISC adaptor protein FADD, caspases-8 and -10 play a role in cell proliferation (Imtiyaz et al., 2009). Peripheral T-cells from FADD deficient mice show a profound impairment of proliferation once they are activated by mitogens or antigens, leading to a reduced number of mature T-cells (Zhang et al., 1998). This inhibition of proliferation results from a failure to enter the cell cycle at the beginning of S-phase due to abnormal expression of cyclin-dependent kinases (Zhang et al., 2001). Pharmacological inhibition of caspases prevents T-cell proliferation *in vitro* supporting the idea that caspase activity is required for proliferation (Kennedy et al., 1999). The cell cycle role for caspases may not be limited to T-cells as impaired T-cell, B-cell, and NK-cell proliferation is seen in immune-deficient humans with caspase-8 defects (Chun et al., 2002).

As well as caspases-8 and -10, caspase-3 has also been found to play a role in regulating cell proliferation. Proliferative brain cells were found to contain active caspase-3, localized in the nucleus (Oomman et al., 2005). In lymphoid cells, caspase-3 supports the proliferation through cleavage of the CDK inhibitor p27 (Frost et al., 2001). In these examples, caspase-mediated stimulation of proliferation appears to be a cell-autonomous event. On the other hand, proliferating cells utilize caspases in a non-apoptotic capacity to downregulate cell cycle inhibitors which normally keep the cell in a quiescent state (Zhang et al., 2001; Woo et al., 2003; Lamkanfi et al., 2007). For example, caspase-3 can exert a strongly anti-proliferative effect in B-cells through cleavage of p21 and caspase-3 knockout mice show a hyperproliferative phenotype in their B-cells (Woo et al., 2003). Thus, killer caspases seem to be able to exert both positive and negative regulation of cell proliferation through selective cleavage of cell cycle regulators without necessarily inducing apoptosis.

Caspase-1, which is involved in toxin-sensing (Li et al., 1995; Franchi et al., 2009), and caspase-11, involved in the production of inflammatory factors (Kayagaki et al., 2011) have long been associated with inflammation and immunity and are not central to cell death processes. However, it has more recently been discovered that apoptotic caspases also have roles in immunity. This can be through their role in the differentiation programs of immune cells, such as with caspase-8 paralog Dredd (Leulier et al., 2000), or through the modulation of the immune response itself, such as with caspase-12 (Saleh et al., 2004) and caspase-3 paralog ced-3 (Aballay and Ausubel, 2001). Such modulation may occur through the generation of inflammatory and anti-inflammatory factors (Kuranaga and Miura, 2007) or through their role in the apoptosis of infected cells.

## Differentiation

Caspases engage in irreversible signal transduction (Kuranaga, 2012). Such irreversible signaling mechanisms are suitable for guiding cell fate choices, such as differentiation. Indeed, such caspase signaling has been found to play important roles in the terminal differentiation programs of several cell types, both in early development, and in tissue regeneration.

The first cell types in which caspases were found to have a direct role in differentiation had one feature in common: their differentiation programs bore a strong resemblance to apoptosis. For example, during terminal differentiation of the lens fiber cells degenerative processes including organelle degradation, chromatin condensation, and DNA fragmentation all occur, and are mediated by the activity of caspases (Ishizaki et al., 1998). The time required for this apoptosis-like process is much longer than that required for caspase-driven cell death, suggesting a more controlled and meticulous version of the same general procedure. Soon after this, erythrocytes and keratinocytes were also found to utilize caspase activity in their terminal differentiation programs (Eckhart et al., 2000; Zermati et al., 2001).

Subsequently, it was found that caspases also play roles in differentiation programs that bore no major similarity to apoptosis. An example of this is the differentiation of peripheral blood monocytes into macrophages, which requires the activation of the caspases-3, -8, and -9 for differentiation (Sordet et al., 2002). Deletion of caspase-3 limits the cytokine-induced differentiation of hematopoietic stem cells (Janzen et al., 2008) and the differentiation ability of iPSCs is enhanced by transient induction of caspase activity (Li et al., 2010b). Several other caspase-dependent cell differentiation programs have been discovered, including those of skeletal myoblasts, osteoblasts, spermatids, placental trophoblast, and embryonic and neural stem cells (Table 2).

**Table 2:** **Caspase involvement in the differentiation programs of several cell types**.

**Differentiation morphology**	**Cell type**	**Caspase(s)**	**References**
Apoptosis-like	Erythrocyte	Caspase-2, -3, -9	Zermati et al., 2001
	Keratinocyte	Caspase-3, -14	Weil et al., 1999; Eckhart et al., 2000
	Epithelial lens	Caspase-3	Ishizaki et al., 1998
	Megakaryocyte	Caspase-3, -9	De Botton et al., 2002
Non-apoptosis-like	Macrophage	Caspase-3, -8, -9	Sordet et al., 2002; Kang and Ben-Moshe, 2004
	Skeletal myoblast	Caspase-3, -9	Fernando and Kelly, 2002; Murray et al., 2008; Larsen et al., 2010
	Neuron	Caspase-3, -1	Fernando et al., 2005; Vaisid et al., 2005
	Glial cell	Caspase-3	Oomman et al., 2006
	Osteoblast	Caspase-2, -3, -8	Mogi and Togari, 2003
	Placental trophoblast	Caspase-8	Black et al., 2004
	Embryonic stem cell	Caspase-3	Fujita et al., 2008
	Hematopoietic stem cell	Caspase-3	Janzen et al., 2008
	Spermatid	Drice (Caspase-3), Dredd (Caspase-8), Dronc (Caspase-9)	Arama et al., 2003, 2007; Huh et al., 2004; Kaplan et al., 2010
	Odontoblast	Caspase-7	Matalova et al., 2013

Some programs follow a “frustrated apoptosis” phenotype, while others have a distinctly non-apoptotic-like morphology.

A well-studied example of caspase-stimulated *in vitro* differentiation is that of mouse muscle myoblasts into multinucleated myotubes (Fernando and Kelly, 2002; Murray et al., 2008; Larsen et al., 2010). In this model capase-3-mediates activation in differentiating mouse myoblasts of a specific DNase called CAD (Larsen et al., 2010). Normally CAD (DFF40 in humans) is bound to a chaperone called ICAD (DFF45) that inhibits the nuclease activity of CAD. Caspase-3 mediated cleavage of ICAD releases CAD, allowing it to cleave DNA. This is a key step in the generation of oligonucleosomal DNA fragments seen in apoptosis. Perhaps surprisingly, activation of CAD occurs in differentiating myoblasts and RNAi directed against CAD causes profound inhibition of myoblast differentiation. Larsen et al. propose that CAD-dependent activation of p21 expression is the key event explaining this defect as p21 expression is an early and necessary event in myoblast differentiation (Larsen et al., 2010). In other words, it is proposed that caspase-3 drives non-apoptotic outcomes by inducing expression of specific genes. In addition, Fernando et al. showed that microinjection of active caspase-3 induced expression of muscle specific genes (Fernando and Kelly, 2002). These two reports support the idea that caspase activity is present in the differentiating myoblast (the direct/autonomous model). Caspase-3 activation during differentiation requires caspase-9 and is blocked by overexpression of Bcl-XL (Murray et al., 2008), which implicates the intrinsic or mitochondrial apoptotic pathway, although the role of cytochrome *c* release or Apaf-1 in this differentiation has not been conclusively demonstrated.

Although this seems like strong evidence for cell-autonomous model of caspase action, there are also data to support non-autonoumous roles for caspase activity in muscle differentiation. It has been found that myoblast fusion is driven by apoptotic cells through a phosphatidylserine-mediated activation of the BAI1 receptor. During apoptosis, caspase-dependent presentation of phosphatidylserine (PS) on the surface of dying cells is an important “eat me” signal for phagocytes and so plays a central role in the clearance of apoptotic bodies. Adding apoptotic cells to cultures where caspase activity has been abolished with pharmacological inhibitors restores myoblast fusion, and adding annexin V, a PS-binding protein, blocks myoblast fusion (Hochreiter-Hufford et al., 2013). This finding thus supports the non-cell-autonomous model, in that fusion is driven by caspase-mediated presentation of cell-surface signaling factors on apoptotic cells. It may be that both cell autonomous and non-autonomous roles for caspases are important in myoblast differentiation.

A question that arises with the cell-autonomous model of caspase activity; how is this activity prevented from progressing to apoptosis? The convention is that activation of apoptotic caspases is an irreversible threshold event, leading to a runaway process of proteolytic cleavage, culminating in apoptotic cell death. If caspases truly are activated within differentiating cells themselves, there must be some mechanism for restraining, sequestering, or otherwise preventing this activity from killing the cell. Members of the IAP family are important caspase regulators (Mace et al., 2010), but there is so far little evidence that any of these proteins regulates caspase activity during muscle differentiation. Kaplan et al. showed that in spermatids there is a gradient of the giant IAP protein, dBRUCE, that establishes a gradient of caspase activity during the process of spermatid individualization in *Drosophila* (Kaplan et al., 2010). There is also other evidence for caspase localization being important during differentiation (Table 3). Interestingly, myogenin expressing satellite cells from young donors display active caspase-3 only at the nucleus, whereas myogenin expressing satellite cells from aged donors contain active caspase-3 both at the nucleus and at the cytoplasm. The satellite cells from aged donors also show a higher level of apoptotic cell death and together these data suggest a model in which the failure to properly localize active caspase-3 leads to satellite cell death and impaired muscle regeneration as we age (Fulle et al., 2013).

**Table 3:** **Compartmentalization of caspases has been found to be used in the differentiation programs of several cell types**.

**Cell type**	**Caspase**	**Form of compartmentalization**
Platelet	Caspase-3	During platelet formation, caspase activity is localized in punctate bodies within the cytoplasm (De Botton et al., 2002)
Lens cell	Caspase-3	Partial localization to equatorial epithelium (Weber and Menko, 2005)
Spermatid	Drice (Caspase-3)	Localized within the cystic bulge of the cytoplasm (Kaplan et al., 2010)
Keratinocyte	Caspase-3	Probable localization in the nucleus during enucleation (Okuyama et al., 2004)
Glia	Caspase-3	Active caspase localized to the nucleus (Oomman et al., 2005, 2006)

Moving to *in vivo* models of muscle differentiation, the role of caspases in regeneration becomes less clear. In caspase-9 (Hakem et al., 1998; Kuida et al., 1998) and caspase-3 (Kuida et al., 1996) knock-out mice embryonic myogenesis appears normal so the role of caspase-9 and caspase-3 in muscle differentiation *in vivo* at first appears unlikely. However, besides prenatal myogenesis, there is a distinct postnatal muscle development process as well as repair and regeneration processes in adult muscle that have not been evaluated in the caspase knock-out mice. Defects in these processes underlie a range of muscular dystrophies and age-related sarcopenia. In some instances, defects that have profound effects on muscle regeneration do not affect embryonic muscle development. For example, mice lacking caveolin-3 or expressing a Pro104Leu mutation in caveolin-3 (a model for human Limb Girdle Muscular Dystrophy 1C) show normal muscle development but muscle degeneration after 8 weeks of age (Hagiwara et al., 2000; Sunada et al., 2001). It is therefore possible that caspase-driven processes are important primarily in regeneration of adult muscle rather than muscle development but defects have not been observed in caspase deficient mice because of the perinatal lethality associated with these knock outs.

Activation of caspase-8 by TNF induces apoptosis and blocks muscle regeneration in *in vivo* models of cachexia (Moresi et al., 2008, 2009), data that also appears inconsistent with a model in which caspase activity is required for differentiation. The conflicting reports of the role of caspases in muscle differentiation may be reconciled by a model in which caspase-8 induces high levels of effector caspase activity that kill cells while differentiation is associated with lower effector caspase activity. Just such a switch between death and differentiation has been reported in Drosophila models (Florentin and Arama, 2012). Alternatively, TNF-dependent caspase activation may result in a different localization of active caspases compared to caspase activation associated with differentiation as discussed above for young and aged satellite cells (Fulle et al., 2013).

It is also possible that a particular cellular differentiation process involves more than one caspase-driven step. Muscle differentiation may represent an example of this, with caspase signaling from apoptotic cells as well as caspase activity in the differentiating cells. Differentiating myoblasts may even rely on their caspase activity to drive more than one process during differentiation. Larsen et al. present compelling evidence that caspase-3 mediated DNA damage drives changes in gene expression that are required for myoblast differentiation (Larsen et al., 2010). Others have argued that primary consequence of preventing caspase activation in differentiation is a failure of myoblast fusion (Murray et al., 2008). It is possible that caspases contribute to myoblast fusion by influencing cell motility, as this is required for both muscle development (Brand-Saberi et al., 1996; Molkentin and Olson, 1996) and regeneration (Seale and Rudnicki, 2000).

## Motility and metastasis

Cellular locomotion essentially involves the continuous deformation and manipulation of the cytoskeleton to achieve movement. Caspases are the major manipulators of cytoskeletal structure during apoptosis, so it is conceivable that caspases could also have a role in enabling cell motility. In support of this model, *in vitro* studies have shown that caspase-8-knockout mouse embryonic fibroblasts are both motility-defective, and unable to form proper lamellipodia (Helfer et al., 2006). It is thought that caspase-8 engages in a multiprotein complex with calpain to cleave focal adhesion substrates (Helfer et al., 2006).

Additionally, the embryonic lethality of caspase-8 homozygous knockout mice has been attributed to the failure to develop a functional circulatory system through a defect in endothelial cell migration (Kang and Ben-Moshe, 2004). It is as yet unknown how caspase-8 mediates migration. It could be mediated through activation of downstream effector caspases like caspase-3, leading to modification of the cytoskeleton, or it could act through a separate pathway that does not involve executioner caspases. There is even evidence suggesting that the catalytic activity of caspase-8 is not required for its effects on cell motility (Senft et al., 2007). In addition to caspase-8, caspase-3 has also been implicated in cell motility. Pharmacological inhibition of caspase-3 activity reduces cancer cell motility and invasiveness (Gdynia et al., 2007).

Metastasis of tumor cells involves cellular migration and invasion of tissues, and the subsequent growth of secondary tumors at distant sites. Normally, cells are unable to escape into systemic circulation, as detachment from their basement matrix induces cell death through anoikis or amorphosis (Mehlen and Puisieux, 2006). However, when apoptosis is compromised through silencing of the downstream effector caspase-3, caspase-8 can act to promote metastasis. In this state, caspase-8 enters into a complex with FAK and CPN2, engaging a signaling pathway which induces cell migration (Barbero et al., 2009).

**Figure 3:**
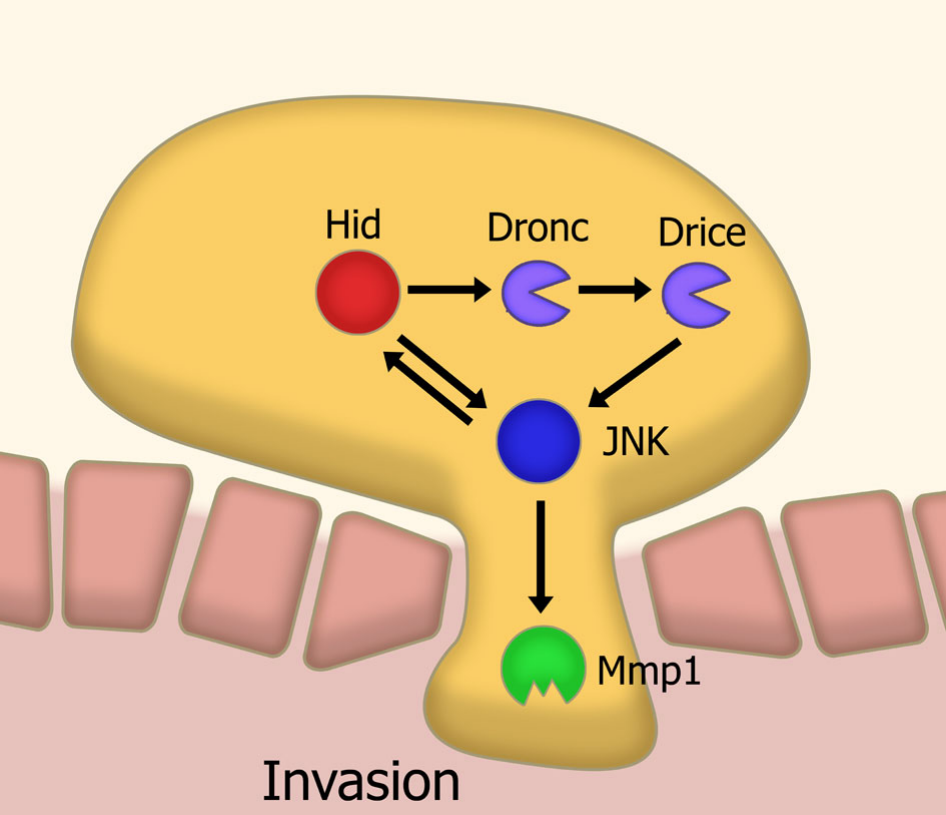
**Model of caspase-mediated tissue invasion based on *Drosophila* studies.** A sub-apoptotic level of caspase activation leads to the activation, via JNK signaling, of matrix metalloproteases. This metalloprotease activity is a necessary step in the invasion of tissues. Abbreviations: JNK, Jun kinase; Mmp1, Matrix metalloprotease 1; Hid, *head involution defective*; Dronc, Drosophila Nedd2-like caspase; Drice, Drosophila ICE.

In a *Drosophila* model of tumor invasion, a non-apoptotic effector caspase pathway is utilized to activate the key invasion protein Mmp1 via JNK signaling (Rudrapatna et al., 2013) (Figure 3). It has been proposed that this cell invasion is achieved through co-opting functions of apoptotic caspase such as cytoskeletal modification. This could be extrapolated as a general feature of non-apoptotic caspase activities in different processes. Together, this suggests a new way of thinking about caspases. Perhaps it is more constructive to think of caspases as cell-structure modifying enzymes rather than as just cell death effectors. This idea is consistent with emerging data showing the role of caspases in neuronal plasticity.

## Neural signaling and potentiation

During early development, live imaging of caspase activity in the brain shows a complex pattern of expression and subcellular localization, occurring in discrete waves (Oomman et al., 2006). These waves of activity correspond to specific periods of brain maturation. Here, we look at the roles of this non-apoptotic caspase activity in neuronal network pruning, synaptic plasticity, signal modulation, and axonal guidance (Hyman and Yuan, 2012).

**Figure 4:**
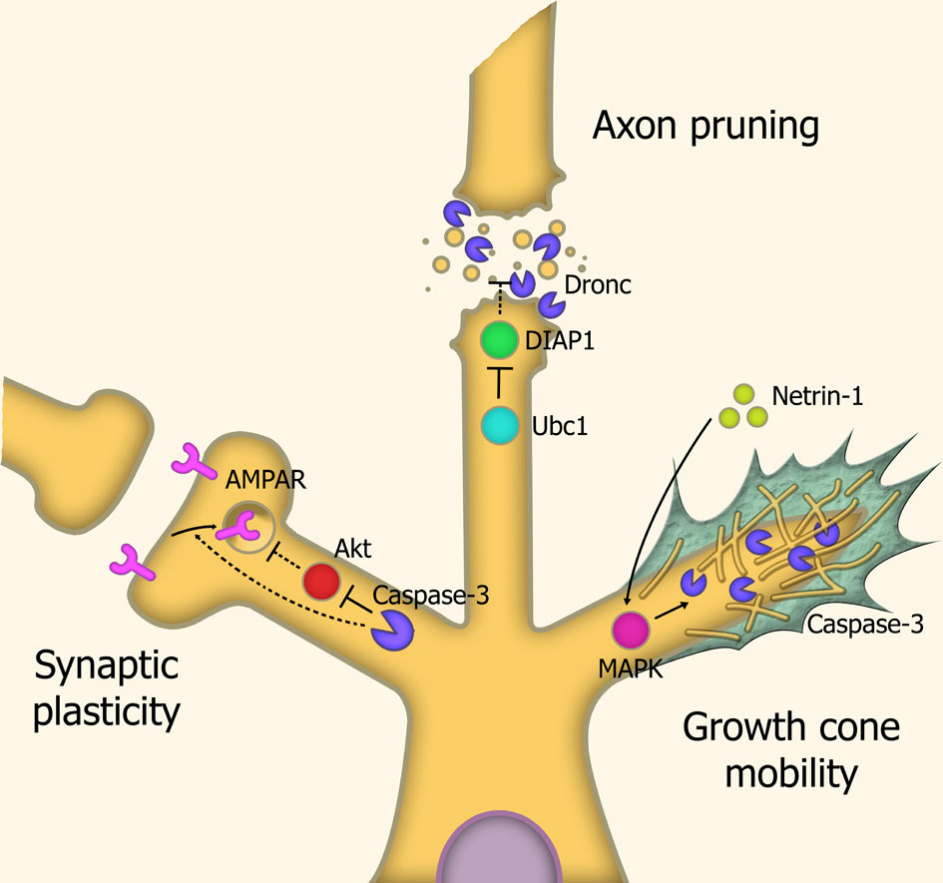
**Caspases in neuronal restructuring and signaling.** *Synaptic plasticity*: Through cleavage of Akt, caspase-3 is involved in the endocytosis of AMPA receptors, leading to Long-Term Depression (LTD) of synaptic signaling. *Axonal pruning:* Localized proteosomal degradation of Inhibitor of Apoptosis proteins (IAPs) within axons lead to a localized caspase activity which shears the axon from the cell body. This process does not kill the parent neuron. *Growth cone mobility:* Netrin-1 acts as a chemoattractant, signaling MAP Kinase-mediated activation of caspase-3, which remodels the cytoskeletal structure within the growth cone, allowing axonal migration down the chemotrophic gradient. Abbreviations: AMPAR, AMPA receptor; Ubc1, Ubiquitin-conjugating enzyme E2 1; DIAP1, Drosophila Inhibitor of Apoptosis 1; MAPK, Mitogen-activated protein kinase.

Pruning of axons and dendrites are the mechanisms through which undesired neural connections are removed. Neural network pruning during larval development in *Drosophila* is carried out through severing the connection between the outgrowth and the cell body, by means of localized executioner caspase activity, which is mediated by the spatially-restricted degradation of IAP proteins through caspase-3-like activity. An essential step in the process is degradation of DIAP1, a key inhibitor of caspase activity. Inhibition of the caspase-3-ortholog Dronc prevents this pruning process (Kuo et al., 2006) (Figure 4).

In mammals, caspases were found to modulate synaptic plasticity through localized activation within synaptic terminals and neurites in response to stressors. Caspase-3 activity leads to dephosphorylation and internalization of AMPA-type receptors. The loss of these receptors causes degradation of the local dendritic spine. This leads to overall modulation of glutamate signaling. Consequentially, caspases have a role in long-term depression (LTD) of neurons, and overexpression of the anti-apoptotic proteins XIAP or Bcl-xL prevent this LTD (Li et al., 2010c).

Axonal guidance is carried out through the diffusion of molecular signals by the target site, generating a chemotrophic gradient for the axon. Caspases also contribute to this chemotrophic migration by regulating the growth of neurites, through localized proteolytic activity within growth cone structures. Caspase-3 activation is required for this response, as LPA-induced growth cone collapse and netrin-1-induced growth cone attraction are both blocked by caspase-3 inhibitors (Campbell and Holt, 2003). This caspase-3-mediated effect does not require caspase-9 activation, suggesting a distinct, non-canonical activation pathway. It has been speculated that caspase-3 mediated modulation of growth cones is carried out through degradation of cytoskeletal structural elements such as actin and gelsolin (Campbell and Holt, 2003) and rock-1 (Riento and Ridley, 2003).

Such caspase-mediated modulation of synaptic plasticity, axon pruning, and growth cone mobility appear to be cell-autonomous events, in that all utilize localized caspase activity within the target cell, likely through spatially-restricted degradation of inhibitors of caspase activity.

## Conclusion

Here, we reviewed the major non-apoptotic roles of caspases discovered to date. We discussed such roles in terms of different cell behaviors such as differentiation, migration, and cell signaling, and presented evidence for the cell autonomous and non-cell-autonomous models of caspase signaling.

In some systems, it seems rather clear that a cell autonomous event is occurring. This is the case in, for example, axonal pruning, where a defined cell autonomous pathway of caspase activation has been elucidated. Other systems appear to be examples of the non-cell-autonomous model. An example of this is the process of compensatory proliferation, whereby caspase-generated signals from apoptotic cells stimulate the proliferation of nearby cells in an intercellular, receptor mediated fashion. Finally, there are systems where the evidence is conflicting. This includes the process of myoblast differentiation, in which there appears to be an essential role for both cell membrane contact with apoptotic, PS exposing cells, and for the cell autonomous caspase activation of nucleases to enable the transcription of myogenic genes. Some further approaches that may prove fruitful for this field include the live imaging of caspase activity in individual cells undergoing differentiation, the identification of soluble mitogenic signaling factors from apoptotic cells, and investigation of the interplay between caspase signaling pathways and other signaling pathways.

Regardless of how caspases are regulated in these models, it seems clear that caspases do indeed have roles other than as effectors of cell death. This new understanding suggests unexpected complications in situations where caspase-dependent cell death is considered desitable, such as in response to cancer chemotherapy. The newfound alternative roles of caspases present the possibility that chemotherapy drugs may induce a wide range of cell behaviors such as increased migration and compensatory proliferation of cancer cells (Jäger and Zwacka, 2010) that are both unexpected and unwanted.

### Conflict of interest statement

The authors declare that the research was conducted in the absence of any commercial or financial relationships that could be construed as a potential conflict of interest.
